# Study on the Irradiation Modification of Reed Straw and the Preparation of Highly Absorbent Gels by Graft Copolymerization

**DOI:** 10.3390/gels12070572

**Published:** 2026-06-29

**Authors:** Jun Guo, Wanrong Li, Na Su, Muhammad Usman, Xingtao Zhang, Lipeng Wu

**Affiliations:** 1 College of Biology and Food Engineering, Suzhou University, Suzhou 234000, China; guojun@ahszu.edu.cn (J.G.); wanrong@ahszu.edu.cn (W.L.);; 2 General College, Anyang University, Anyang 14028, Republic of Korea; 3 Department of Plant Sciences, North Dakota State University, Fargo, ND 58108, USA

**Keywords:** irradiated reed straw, graft copolymerization, high water-absorbing gels, cellulose extraction, water retention properties

## Abstract

The goal of synthesizing more environmentally friendly high-water-absorbing gels (HWAGs) is to reduce the consumption of petroleum resources. We also aimed to make rational use of plant straw. Reed straw (RS) was modified using strong irradiation. Acrylamide (AM) and acrylic acid (AA) were grafted onto the cellulose for graft copolymerization reactions. The cellulose and gels were analyzed using infrared spectroscopy (IR), scanning electron microscopy (SEM), and thermogravimetric (TG) analysis. To reduce the crystallinity of the RS, the optimal irradiation dosage is 96 kGy. Single-factor and orthogonal experiments were conducted to determine the optimal reaction conditions for IRSC-HWAG: the monomer ratio m(AA):m(AM) was 1.5; The material ratio of (m_(AA+Am)_:m_(IRSC)_) was 9:1; The neutralization degree of AA was approximately 90%; The irradiation dose was 5 kGy, with a dose rate of 2.0 kGy/h; the crosslinking agent dosage was 1.2%. The absorption rate of deionized water (Q_d_) by the gels was approximately 1160 g/g; the absorption rate of salt water (Q_s_) by the gels was approximately 99 g/g. A water-retention experiment demonstrated the IRSC-HWAG’s superior water-retention properties. These properties were compared with those of resins synthesized using pure chemical reagents. This experiment provides valuable reference data for the further development of plant-based water-retention agents.

## 1. Introduction

High-water-absorbing gels (HWAG) are colloidal substances with a three-dimensional network structure and a large number of hydrophilic groups [1,2,3]. They are usually used for hygiene products, such as napkins, sanitary pads, medical tourniquets, agricultural water-retaining agents, etc. HWAGs are generally classified into chemical synthesis and biogenic graft–copolymer types [4]. Chemical synthesis products usually cause pollution, high energy consumption, and other problems, while biogenic material products usually offer advantages such as reducing oil consumption, lowering pollution, and being renewable. Bio-based high-water-absorbing gels combine the sustainability of natural materials with the high performance of synthetic materials, and are an important development direction for future functional polymer materials [5,6,7,8]. In our study, we replace traditional petroleum-based products and promote the development of the green materials industry. These materials exhibit an extraordinary capacity for fluid absorption, and are capable of retaining quantities of water or saline solutions ranging from hundreds to thousands of times their own dry mass. Upon hydration, they undergo a transition to a gel state characterized by exceptional water-retention properties. Notably, they demonstrate resilience against dehydration even under compressive forces, while enabling the gradual release of entrapped substances such as fertilizers or therapeutic agents. Derived from natural precursors, these materials possess favorable biocompatibility, facilitating their eventual biodegradation by environmental microorganisms and thereby mitigating their ecological impact.

Natural polymer framework materials typically possess a fundamental biopolymer scaffold, such as cellulose, starch, or proteins, which incorporates a multitude of hydroxyl (-OH), amino (-NH_2_), or carboxyl (-COOH) functional groups, conferring pronounced hydrophilic characteristics [9,10,11]. Through chemical cross-linking methodologies employing agents like epichlorohydrin or citric acid or via physical interactions, a three-dimensional network architecture is established, enabling these gels to imbibe water and swell volumetrically while maintaining their structural integrity without dissolution. Graft copolymerization techniques, particularly when conducted with monomers such as acrylic acid and acrylamide, are frequently employed to introduce additional hydrophilic moieties, thereby further augmenting the water absorption capacity. These materials exhibit notable resistance to dehydration under applied pressure and facilitate the gradual release of water or incorporated active agents, including fertilizers and pharmaceutical compounds. The utilization of naturally derived raw materials imparts excellent biocompatibility, enabling their biodegradation by environmental microorganisms and consequently mitigating their potential ecological impact [6,12].

Plant straw cellulose exhibits a highly crystalline network structure, wherein cellulose molecular chains are arranged in a remarkably ordered, densely packed crystalline configuration, which is particularly evident in cellulose type I, through an extensive network of intramolecular and intermolecular hydrogen bonds. This intricate structural arrangement impedes the penetration and disruption of the crystal lattice by solvent molecules, thereby conferring significant resistance to dissolution [13,14]. The molecular architecture is further characterized by a robust hydrogen-bond network: the copious hydroxyl groups distributed along the cellulose chains facilitate the formation of a three-dimensional hydrogen-bonding system that generates substantial cohesive energy, effectively binding the molecular chains in intimate proximity. Consequently, the dissolution of such a structurally coherent system necessitates that thermodynamically prohibitive energy barriers are overcome [15]. It also has a high degree of polymerization and molecular rigidity. The degree of polymerization (DP) of natural cellulose can reach several thousand or even tens of thousands [16]. The long, rigid molecular chains are difficult to move in solution and are highly entangled, resulting in extremely high dissolution viscosity and even gel formation rather than a true solution. The formed molecular chains are in an extended ribbon-like structure, have a rigidity of β–1,4–glycosidic bonds, and are not easily bent or solvated [17]. Cellulose possesses poor intermolecular affinity; it is a highly polar polymer, but is insoluble in water and hardly soluble in common organic solvents. It is neither a typical hydrophilic substance (because its crystal structure is too strong) nor a hydrophobic substance, violating the “like dissolves like” principle [14,18].

Potent ionizing irradiation encompassing high-energy emissions such as gamma rays or electron beams constitutes a sophisticated physical pretreatment methodology. This approach harnesses high-energy radiation to intimately interact with the structural constituents of straw, thereby inducing a cascade of physicochemical alterations that substantially facilitate subsequent biological or chemical transformations. When subjected to such high-energy ionizing radiation, whether emanating from ^60^Co gamma sources or electron beams generated by particle accelerators, the straw undergoes molecular perturbations primarily through two distinct mechanisms: high-energy photons or electrons directly impinge upon cellulose molecular chains, transferring substantial energy to these macromolecules and precipitating the scission of chemical bonds including but not limited to C–O–C glycosidic linkages, C–H bonds, and C–C bonds, which consequently generates free radicals, ions, and other reactive intermediates of considerable chemical reactivity [19,20,21]. The radiation interacts with water molecules in the straw, generating a large number of highly reactive •OH (hydroxyl radicals), •H (hydrogen radicals), hydrated electrons (^e−a^q), etc. [22,23]. These reactive species subsequently initiate an assault upon cellulose, hemicellulose, and lignin macromolecules, precipitating a cascade of degradation reactions. Foremost among these effects is the substantial reduction in cellulose’s degree of polymerization (DP), as radiation preferentially targets the glycosidic linkages within extended cellulose chains, thereby cleaving the molecular backbone and diminishing both molecular weight and polymerization indices. This phenomenon represents the most significant and direct consequence of irradiation, effectively fragmenting recalcitrant, high-polymerization-degree native cellulose into more manageable, shorter-chain segments [24]. Secondly, high-energy radiation induces disruption of the crystalline lattice. This irradiation compromises the well-ordered hydrogen-bonding network within cellulose microfibrils, consequently diminishing crystallinity and facilitating the partial conversion of crystalline domains into amorphous regions. These amorphous zones exhibit enhanced porosity, rendering them more susceptible to penetration and subsequent degradation by solvents, enzymatic agents, or chemical reagents. Moreover, such irradiation may engender the formation of novel oxygen-containing functional moieties including carbonyl and carboxyl groups, which modifies the surface chemical characteristics of cellulose, thereby augmenting its hydrophilic nature and chemical reactivity [25]. The irradiation-mediated degradation of crop straw cellulose represents a pristine, efficacious, and physically driven pretreatment methodology, distinguished by its unique mechanism [26]. This process facilitates subsequent dissolution in media such as ionic liquids or alkaline/urea systems under milder operational parameters, including reduced temperature and processing time. Reed straw exhibits a cellulose content typically ranging from 40% to 50%, surpassing that of numerous annual crop residues including wheat straw (approximately 35–40%) and rice straw (approximately 32–37%) [12]. Consequently, a greater quantity of purified cellulose can be extracted per unit mass of raw material, thereby enhancing overall resource utilization efficiency [27]. The fiber morphology of reed straw, characterized by moderate fiber length and aspect ratio, demonstrates superior structural integrity compared to certain other lignocellulosic grasses. Furthermore, its diminished lignin and silica content contributes to a less recalcitrant cellular structure, thereby improving processability. Thanks to the high purity of cellulose and the low impurities, the cellulose from reed after dissolution is very suitable for preparing high-value-added bio-based functional materials [28].

This study obtained cellulose with improved solubility by pre-exposing reed straw to strong radiation, and produced high-water-absorbing gels with higher water-absorption capacity by irradiating acrylamide and acrylic acid.

## 2. Results and Discussion

### 2.1. Pre-Treatment Methods for Reed Straw

From Table 1, we know that the cellulose yield obtained from processing RS was the highest for the C6 variety, followed by C5 and C7. The lowest-yielding was C1. The cellulose yield began to increase with irradiation dosage, reaching its peak at 48 h before decreasing.

In contrast to conventional pretreatment methodologies, this approach facilitates efficient depolymerization and the structural remodeling of cellulose under ambient temperature and pressure conditions. The process directly reduces the degree of polymerization and crystallinity without necessitating chemical reagents, thereby fundamentally altering the recalcitrant native structure of cellulose. Characterized as a “dry processing” technique, this method offers substantial environmental advantages by eliminating wastewater and solid waste generation, particularly in contrast to chemical pretreatment, which produces substantial quantities of black liquor while ensuring the absence of chemical residues. Furthermore, the process requires minimal post-treatment procedures. Notably, it demonstrates remarkable homogenization effects, exhibiting profound penetration capabilities (especially with gamma irradiation), enabling the processing of substantial volumes and irregularly shaped raw materials while achieving uniformly distributed treatment outcomes.

The procedure is characterized by its operational simplicity and precise controllability, enabling the modulation of degradation extent through meticulous adjustment of the irradiation dose, a critical parameter typically ranging from 10 to 100 kGy. This technique exhibits remarkable compatibility with subsequent processing stages, including enzymatic hydrolysis and dissolution protocols. According to the data presented in Table 1, the LiCl/DMSO solvent system demonstrates superior cellulose production yields compared to the urea/sodium hydroxide alternative. The LiCl/DMSO system generally possesses enhanced cellulose dissolution capabilities, facilitating the complete solubilization of the native cellulose substrate. Moreover, during the regeneration phase, this system allows for greater ease in obtaining uniformly regenerated materials. Theoretically, under optimized processing conditions, this methodology can potentially yield higher production/recovery rates or produce materials of enhanced quality, exemplified by superior initial retention and mechanical properties in membranes, fibers, and sheets.

Urea/sodium hydroxide systems, exemplified by the cold urea/NaOH configuration, represent an early generation of cellulose dissolution methodologies operating under mild thermal conditions. This approach is characterized by its operational simplicity and reduced corrosive potential toward processing equipment. Nevertheless, the system exhibits limitations in solvation capacity and demonstrates a narrower spectrum of molecular weights for cellulose dissolution compared to the LiCl/DMSO alternative. Furthermore, stringent temperature regulation and expeditious processing are imperative to prevent cellulose degradation. While the LiCl/DMSO system may demonstrate superior outcomes in terms of yield and product quality under certain conditions, the urea/NaOH approach presents greater feasibility for cost-effective manufacturing and industrial scale up. As delineated in Table 2, cellulose samples C1 through C8 correspond, respectively, to hardwood acetylated glucans (HWAGs) HWAG1 through HWAG8. Among these, HWAG6 exhibits the most favorable distilled water absorption ratio. Consequently, HWAG6 was selected for subsequent characterization studies in this investigation.

**Figure 1:**
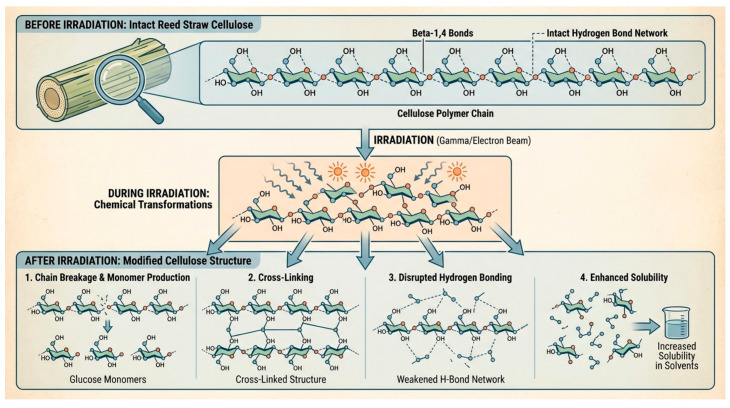
The principle of irradiation-induced degradation of RSC( Generated by ChatGPT).

As depicted in Figure 1, excessive cellulose degradation induces pronounced molecular fragmentation, consequently reducing the molecular weight (degree of polymerization, DP). Nevertheless, this degradation does not invariably correlate with enhanced swelling or hydration properties. Excessive depolymerization can impede the formation of a cohesive network; the segments between truncated polymer chains exhibit a propensity for slippage, thereby precluding the establishment of a stable three-dimensional architecture. This results in an irregular distribution of cross-linking sites, diminished network integrity, and reduced capillary attraction [29]. Furthermore, increased chain scission at the terminal regions diminishes opportunities for intermolecular polymerization during the grafting reaction. An excessive abundance of available chain ends in the degraded products may perturb the distribution of free radicals or active sites, thereby reducing grafting efficiency and yielding insufficient effective cross-linking moieties [30].

Secondly, the reaction kinetics are attenuated. The segmental mobility of the high-molecular-weight polymer diminishes, consequently reducing the reaction rate and resulting in inadequate grafting site density, which impedes the formation of a sufficiently crosslinked network. The increased molecular weight engenders an expanded hydration layer and augmented diffusion resistance within the sol/swelling milieu, thereby fostering a denser “skin” layer while simultaneously impeding water access to the core region. This phenomenon precipitates a diminished overall water absorption expansion rate, with the effect being particularly pronounced in nonionic network systems. Ultimately, the resultant network architecture may exhibit diminished homogeneity in pore size distribution or the concurrent presence of macropores and extensive collapsed domains, thereby compromising water-permeation adsorption kinetics. If the amount of grafting monomer does not match, excess monomers or crosslinking agents will “aggregate” into lumps on the high DP matrix, forming an inhomogeneous network that reduces free volume and water-binding sites at the equilibrium expansion stage [31].

### 2.2. Product Characterization Analysis

#### 2.2.1. Analysis of the Infrared Spectrum

**Figure 2:**
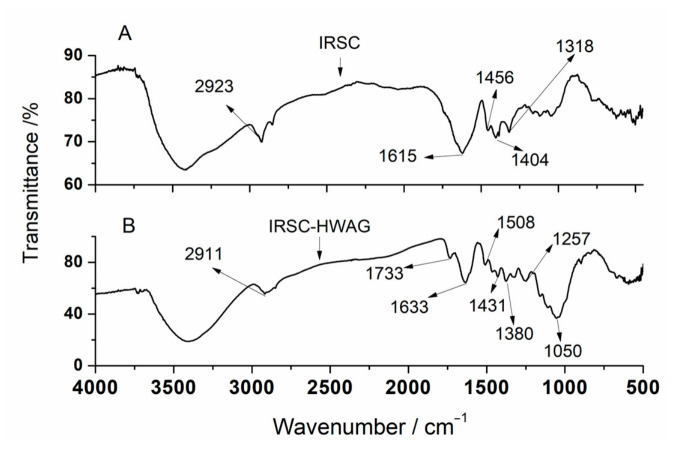
Infrared spectra. (**A**) IRSC; (**B**) IRSC–HWAG.

As illustrated in Figure 2A, the infrared spectroscopic analysis elucidates several salient characteristics of the IRSC. Distinct absorption bands at 2911 and 2923 cm^−1^ correspond to the anti-symmetric stretching vibrations of aliphatic C–H bonds, thereby corroborating the presence of aliphatic chains within the material. A pronounced absorption peak at 1733 cm^−1^ is attributable to C=O stretching vibrations, which are characteristic of carbonyl groups typically found in ester or amide functionalities.

As shown in Figure 2B, with respect to the grafted HWAG specimen, this particular absorption band suggests intermolecular interactions between amide and carboxyl groups, indicative of polyamide-like segments engaging in molecular interactions with the carboxylate functionalities incorporated during the grafting process. The presence of amide-containing moieties is further substantiated by additional absorption bands associated with C=O and N–H vibrational environments. Notably, bands observed at 1404 and 1318 cm^−1^ are assigned to the symmetric and asymmetric stretching modes of the carboxylate (–COO^−^) group, respectively, which are associated with sodium counterions (–COONa). The appearance or enhancement of these bands in the grafted spectrum indicates the formation or stabilization of carboxylate functionalities, possibly due to grafting processes involving carboxylate-bearing monomers or post-grafting hydrolysis/neutralization effects leading to sodium carboxylate formation. The spectral features at 1456 and 1615 cm^−1^ are ascribed to C–O–C stretching vibrations characteristic of cycloether configurations, thereby signifying either the formation or preservation of ether-linked constituents within the grafted copolymer architecture. Pronounced absorption bands at 1431, 1380, 1257, and 1050 cm^−1^ correspond to C–H plane rocking vibrations diagnostic of extended carbon chains, corroborating the substantial aliphatic character of the grafted copolymer and elucidating the contribution of elongated hydrocarbon backbones to the material’s composition [32,33].

#### 2.2.2. Thermogravimetry Analysis

**Figure 3:**
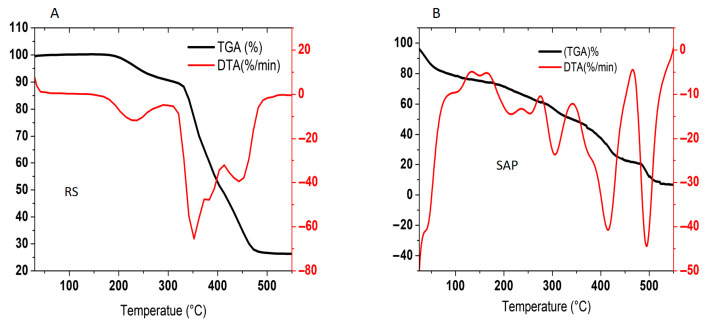
TG and DTA of the sample. (**A**) IRSC; (**B**) IRSC–HWAG.

As shown in Figure 3A, thermal analysis below 190 °C reveals only marginal mass loss, which can be attributed to the presence of residual moisture within the ostensibly dry sample. Conversely, Figure 3B demonstrates that the introduction of water elicits a discernible thermogravimetric response in the resin matrix, with this effect being particularly pronounced below 120 °C. This low-temperature desorption phenomenon corresponds to the volatilization of unbound water molecules entrapped within microvoids and adsorbed at hydrophilic sites present in both the cellulose and resin phases. These observations corroborate the theoretical expectation that residual moisture significantly influences the initial segment of the thermogravimetric curve, thereby potentially compromising the subsequent interpretation of decomposition onset temperatures. Consequently, meticulous sample-drying and baseline correction procedures are imperative for unequivocally distinguishing intrinsic material stability from moisture-induced analytical artifacts. The initial decomposition temperature (T2) thus emerges as a practical parameter for evaluating the onset of substantive thermal degradation.

For IRS cellulose (Figure 3A), the thermal degradation profile, as evidenced by T2 values, spans approximately 310–480 °C, demonstrating the remarkable structural integrity of the sugar ring framework when subjected to thermal stress. This extensive thermal onset range suggests the concurrent occurrence of multiple degradation pathways, including the cleavage of glycosidic linkages and the scission of ring structures, with the overall degradation process concluding by approximately 550 °C. The breadth of this thermal transition window further implies heterogeneity within the cellulose matrix, potentially attributable to variations in substitution patterns, crystallinity indices, and residual moisture content despite prior drying procedures. In contrast, IRS–HWAG (Figure 3B) exhibits a significantly reduced initial decomposition temperature, commencing at approximately 200 °C, while concurrently maintaining a similar degradation completion temperature near 550 °C.

For Figure 3B, the reduced transverse relaxation time (T2) observed for IRS–HWAG suggests an alternative degradation pathway or a less extensively crosslinked matrix in comparison to IRS cellulose. This discrepancy may potentially be attributed to variations in the polymer network architecture, substituent distribution patterns, or the presence of sulfonated species or crosslink-modulating compounds inherent to the IRS–HWAG formulation. Notably, the thermal degradation profile of IRS–HWAG lacks the pronounced rapid-degradation phase characteristic of certain polymeric systems, indicating a more gradual decomposition process without exhibiting a distinct two-stage mass loss phenomenon. Under identical thermal analysis conditions, the resin component demonstrates diminished decomposition kinetics relative to IRS cellulose. This observation implies that the grafting of resin onto the IRS cellulose substrate promotes a more homogeneous dispersion of the grafted monomer along the cellulose macromolecular framework [1,34,35].

#### 2.2.3. SEM of Irradiated Reed Straw–HWAG

**Figure 4:**
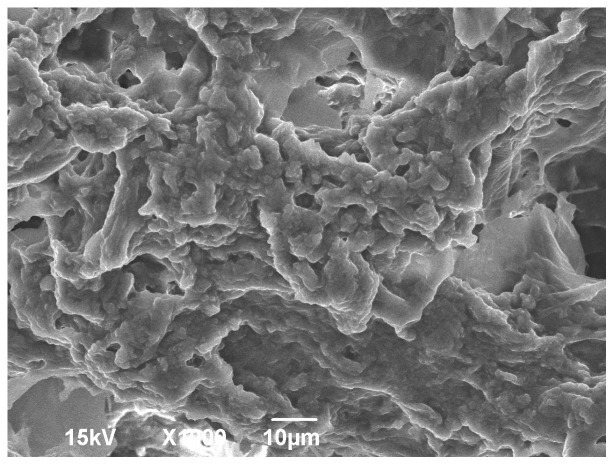
SEM of IRSC–HWAG.

As shown in Figure 4, the IRS–HWAG surface exhibits a distinctly nonuniform morphology, characterized by pronounced topographical features such as ridges, crevices, and nodular protrusions. These irregularities engender a substantial degree of surface roughness, a parameter widely acknowledged to modulate capillary dynamics and water-vapor sorption behavior. The porous architecture spans multiple orders of magnitude, encompassing dimensions from submicron to several micrometers, thereby establishing a hierarchical porosity that facilitates enhanced liquid ingress and retention capacity. This structural heterogeneity may promote rapid initial water uptake through capillary channels while concurrently preserving mechanical integrity during swelling phenomena. The resultant loose, highly porous network architecture correlates strongly with a rapid water absorption kinetics.

The extensive surface area inherent to the irregular, non-smooth morphology of the material provides ample sites for molecular interactions between water and the hydrophilic moieties present within the graft copolymer. The hydrophilic functional groups contained within the copolymer structure are capable of forming hydrogen bonds with water molecules. Upon hydration, these functional groups establish hydrogen-bonding interactions that, through a network of such connections, effectively retain water within the hydrogel structure. The synergistic combination of high surface area, porous interconnectivity, and hydrophilic functionality collectively contributes to the exceptional water absorption capacity observed in this system [3,36,37].

### 2.3. Single-Factor Experiment

#### 2.3.1. Influence of the Ratio of Am to AA on the Water Absorption Capacity of IRSC–HWAG

**Figure 5:**
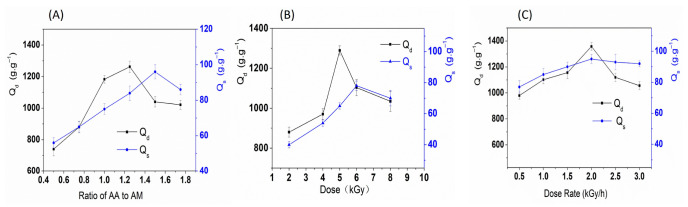
(**A**) Effect of AA-to-AM ratio on the water absorption rate of IRSC–HWAG. (**B**) Effect of radiation dose on the water absorption rate of IRSC-HWAG. (**C**) Effect of radiation dose rate on the water absorption rate of IRSC–HWAG.

The experimental conditions of the single-factor design were set according to the relevant literature [6,15]. The radiation dose rate was 1.5 kGy/h, the irradiation dose was 5 kGy, and the neutralization degree was 90%. Figure 5A presents a systematic analysis of the phenomenon wherein the Q_d_ ratio of the graft copolymer IRSC–HWAG attains its maximum value when the molar ratio of monomer AA to monomer Am reaches 1.3. Additionally, this study examines the pronounced escalation of the Q_s_ ratio at monomer ratios below 1.5, which are followed by a gradual decline upon exceeding this threshold.

From one perspective, the augmentation of AA corresponds to an increased concentration of –COO^−^ groups within the graft copolymer structure, potentially influencing the hydrogen-bonding interactions with water molecules. Conversely, the investigation considers the regulatory influence of solution ionic strength and the presence of electrolytes on water absorption behavior. Furthermore, this study explores how the proliferation of –COO^−^ sites modifies the primary determinants of osmotic pressure generation, thereby altering the coupled relationship between Q_s_ and Q_d_ parameters.

The hydrophilic characteristics of the graft copolymer are governed by the dissociation of carboxylate anions (–COO^−^) and their subsequent hydrogen-bonding interactions with aqueous molecules, coupled with the osmotic pressure-generation mechanism. Consequently, the wettability of the copolymer is contingent not merely upon the total concentration of functional groups but also upon their spatial distribution conformation, conformational mobility, and the ionic composition of the medium. Within the macromolecular architecture of the graft copolymer, the carboxylate groups constitute a pivotal functional moiety, facilitating ionization and mediating interactions with water molecules. An augmentation in the Aa content correspondingly elevates the density of –COO^−^ groups within the copolymer network [38,39].

#### 2.3.2. Influence of Irradiation Dose on Water Absorption Capacity of IRSC-HWAG

The experimental conditions of the single-factor design were set according to the relevant literature [6,15]. The radiation dose rate was 1.5 kGy/h, the irradiation dose was 5 kGy, the neutralization degree was 90%, and the ratio of AA:Am was 1.2:1. As shown in Figure 5B, within the 2–5 kGy irradiation range, the resin’s affinity for deionized water exhibited a pronounced upward trajectory, culminating at 5 kGy with maximal water absorption capacity (Q_d_). This inflection point signifies the attainment of an optimal synergistic state between the resin’s hydrophilic characteristics and its network architecture under this specific dosage regimen. Concurrently, across the 2–6 kGy spectrum, the resin’s absorption capacity (Q_s_) for physiological saline demonstrated a progressive enhancement, attaining its zenith at 6 kGy. This observation suggests that within this dosage interval, the resin’s porous network and ion transport pathways underwent optimal configuration, thereby facilitating enhanced permeation and absorption of the saline solution. Collectively, these findings indicate that the 6 kGy irradiation dosage represents the point of optimal coupling between hydrophilic affinity and network synergism in the studied system. Within the low-to-medium dose range (approximately 2–6 kGy), irradiation-mediated cross-linking and the incorporation of hydrophilic groups tend to augment the resin’s hydrophilicity and network connectivity, thereby enhancing both Q_d_ and Q_s_ values. At the optimal point (maximum Q_d_ at 5 kGy and maximum Q_s_ at 6 kGy), the network effect and hydrophilicity are well-coupled, and water entry and ion transport are synergistically enhanced in both spatial distribution and kinetics [40,41].

#### 2.3.3. Influence of Irradiation Dose Rate on Water Absorption Capacity of IRSC–HWAG

The experimental conditions of the single-factor design were set according to the relevant literature [6,15]. The irradiation dose was 5 kGy, the neutralization degree was 90%, and the ratio of AA:Am was 1.2:1. As shown in Figure 5C, When the irradiation dose rate remains below 2.0 kGy/h, both the degree of grafting (Q_d_) and grafting efficiency (Q_s_) exhibit a positive correlation with increasing dose rate. This phenomenon suggests a progressive enhancement in the coupling efficiency between free radical generation and the subsequent grafting reaction, accompanied by the gradual formation of a more complete network structure. Conversely, upon exceeding the 2.0 kGy/h threshold, Q_d_ and Q_s_ demonstrate an inverse relationship with dose rate elevation. This reversal indicates the predominance of thermal effects and spatial hindrance phenomena, alongside the suppression of homocyclization reactions, a reduction in available grafting sites, and consequent structural inhomogeneity within the network. Collectively, these observations demonstrate that excessively low or high dose rates compromise the product’s water absorption capacity. An optimal dose rate of approximately 2.0 kGy/h should therefore be established to maximize both grafting performance and network integrity. While higher dose rates indeed generate a greater quantity of primary free radicals per unit time, the conversion efficiency of these radicals shows a non-linear relationship with dose rate [22,42,43].

#### 2.3.4. Influence of Neutralization of AA on the Water Absorption Capacity of IRSC-HWAG

**Figure 6:**
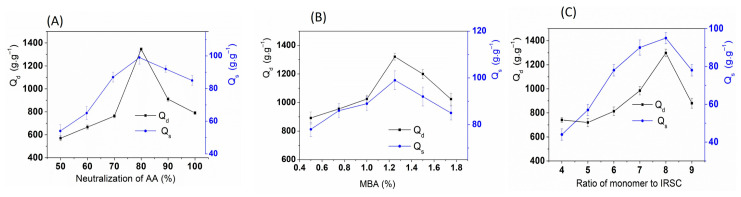
(**A**) effect of neutralization degree of AA on the water absorption rate of IRSC–HWAG. (**B**) Effect of MBA on the water absorption rate of IRSC–HWAG. (**C**) Effect of monomer to IRS ratio on water absorption rate of IRSC–HWAG.

The experimental conditions of the single-factor design were set according to the relevant literature [6,15]. The radiation dose rate was 1.5 kGy/h, the irradiation dose was 5 kGy, the neutralization degree was 90%, and the ratio of AA:Am was 1.2:1. As shown in Figure 6A, the experimental data concerning neutralization degree reveal that the influence of monomer neutralization on water absorption manifests a distinctive “kinked” pattern. The gel’s water absorption capacity attains its maximal value when the monomer neutralization degree approaches approximately 80%. This phenomenon may be elucidated through the following dual mechanisms. Primarily, the neutralization degree governs the ratio of carboxylic acid to carboxylate moieties within the system, thereby directly modulating both the quantity and spatial distribution of hydrophilic functionalities. An augmentation in neutralization degree enhances the density of hydrophilic groups including hydroxyl and carboxylate species within the polymer matrix, consequently facilitating greater water molecule penetration into the network and promoting both swelling and water uptake. Second, an excessively high neutralization degree will have an adverse effect on the formation and stability of the crosslinking network. When the neutralization degree exceeds approximately 80%, the water absorption rate decreases. This reduction in effective polymerization events consequently diminishes the ultimate crosslinking density of the resultant network. Such diminished crosslinking is manifested as compromised gel strength, diminished water resistance, and an increased propensity for resin precipitation or aqueous dissolution [44,45].

#### 2.3.5. Influence of Crosslinker on Water Absorption Capacity of IRSC–HWAG

The experimental conditions of the single-factor design were set according to the relevant literature [6,15]. The radiation dose rate was 1.5 kGy/h, the irradiation dose was 5 kGy, the neutralization degree was 90%, and the ratio of AA:Am was 1.2:1. The trend depicted in Figure 6B reveals that increasing MBA concentration induces a distinctive curve in the product’s water absorption capacity, characterized by an initial ascent followed by a subsequent decline. Specifically, when MBA utilization remains below 1.2%, the network cross-linking density proves insufficient, resulting in a pronounced augmentation of the grafted copolymer’s hardness subsequent to water absorption. As the cross-linking agent concentration progressively increases, the number of connection points within the network escalates, while the pore and void structures undergo optimization. This enhancement expands the pathways available for water molecule ingress and diffusion throughout the network, thereby increasing the water absorption capacity until the inflection point of 1.2% is reached, at which the maximum value is attained. When the MBA content exceeds 1.2%, the water absorption capacity exhibits a notable decline. Empirical evidence indicates that while an augmentation in cross-linking density typically enhances the mechanical integrity and dimensional stability of hydrophilic polymers, an excessive cross-linking density often compromises the percolation pathways for water molecules, thereby diminishing the overall absorption rate. Consequently, the optimal balance is typically achieved at a methylenebisacrylamide (MBA) concentration of 1.2%, at which the network architecture maintains both effective diffusion channels and a robust structural framework, thereby facilitating superior water absorption and retention capabilities [46,47].

#### 2.3.6. Influence of Monomer-to-IRSC Ratio on Water Absorption Capacity of IRSC–HWAG

The experimental conditions of the single-factor design were set according to the relevant literature [6,15]. The radiation dose rate was 1.5 kGy/h, the irradiation dose was 5 kGy, the neutralization degree was 90%, and the ratio of AA:Am was 1.2:1. As shown in Figure 6C, under controlled conditions where all other parameters remain constant, the systematic variation in raw material composition permits the elucidation of the governing principles by which IRSC-HWAG influences Q_d_ and Q_s_ as a function of monomer proportion. Within an optimally defined monomer concentration range, augmentation of the monomer dosage manifests as a progressive enhancement in both parameters. However, upon exceeding a critical threshold concentration, the adsorptive capacities of Q_d_ and Q_s_ commence a discernible decline. The observed phenomenon may be attributed to the fact that with increasing monomer proportion, the availability of active sites within the IRSC–HWAG system correspondingly increases, thereby elevating the frequency of grafting reactions. Upon reaching a monomer proportion of 8, both Q_d_ and Q_s_ attain their respective maxima. The augmentation of monomer proportion significantly elevates the density of polar groups, thereby enhancing the material’s affinity for water and its permeability characteristics. The increased proportion of polar groups concurrently reduces osmotic pressure, facilitating more facile water penetration and retention within the material, which subsequently enhances the Q_s_ absorption capacity [33,48].

### 2.4. Optimization of the Reaction Parameters on the Water Absorption Capacity of IRSC–HWAG

Employing a four-factor, three-level orthogonal experimental design as our analytical framework, this investigation systematically examines the multifaceted influences of the four principal factors and their respective level combinations on key performance indicators—specifically, the resin’s salt absorption capacity and water absorption rate—with the objective of determining the optimal combination (SPSS v. 19.0, IBM Inc., Armonk, NY, USA). The experimental data and design protocol are comprehensively detailed in Table 3. The four factors and their corresponding levels are delineated as follows: Factor A represents the mass ratio of irradiated reed straw to monomer (m_IRS_:m_m_); Factor B denotes the neutralization degree of acrylic acid; Factor C signifies the radiation dose; and Factor D indicates the mass ratio of acrylic acid to acrylamide (m_AA_:m_Am_, *w*/*w*). Notably, Table 3 elucidates the discrete values of each factor at the three experimental levels, as well as their potential interactive effects. It is noteworthy that under the A3B3C2D2 formulation, the resin exhibits a saline absorption capacity of 99 g/g within a 0.9% mass fraction sodium chloride aqueous solution, whereas its absorption capacity in deionized water reaches 1160 g/g. These findings indicate that in saline environments, elevated concentrations of constituents A and D synergistically augment the saline absorption rate. Conversely, in deionized water, the water absorption performance markedly surpasses that of other formulations, with the A2B2C2D2 combination demonstrating the highest water absorption rate at 1350 g/g. These disparate absorption characteristics provide a critical foundation for selecting modified resins in practical applications, particularly when considering varying environmental conditions.

Furthermore, the statistical and comparative analysis presented in Table 3 reveals a distinct hierarchical pattern in the water absorption rates of the resins when immersed in distilled water, specifically A > D > C > B. This ranking indicates that resins A and D exhibit a substantially greater affinity for water absorption in this environment, followed by C, with B demonstrating the least absorption. This established relationship provides a qualitative framework for comprehending the differential sensitivity of resin hydration behavior across varying environmental conditions. Based on a comprehensive evaluation of all experimental groups, it becomes evident that hydrogels characterized by high water absorption capacity are particularly well-suited for applications within solute-rich environments. Consequently, the optimal formulation is determined to be A3B3C2D2.

### 2.5. Water-Retaining Property

**Figure 7:**
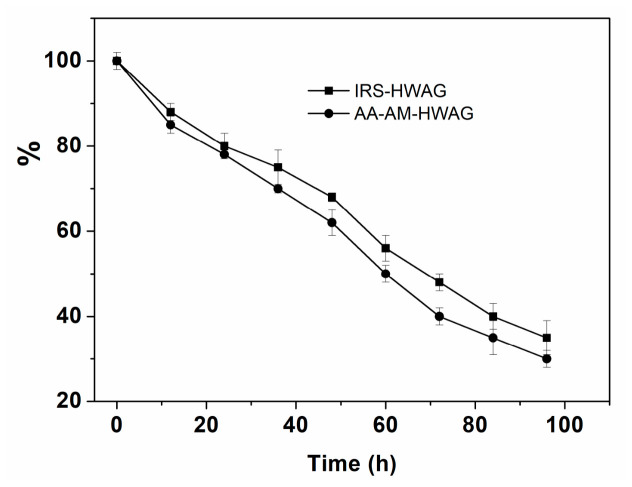
Water-retaining properties of IRS–HWAG and AA–AM–HWAG.

As Figure 7 shown, the experimental findings demonstrate that the IRSC-HWAG system exhibits a substantial initial water absorption capacity in its expanded state, followed by a gradual decline in its moisture retention properties over time. Within the 12 h observation period, this system maintains approximately 87% of its initial moisture content, which decreases to approximately 60% by the 48 h mark. These observations indicate that the network structure possesses remarkable water-retention capabilities. In contrast, the AA-Am-HWAG (AA-Am graft copolymer–gels) demonstrate significantly inferior water-retention characteristics under identical experimental conditions, thereby suggesting that irradiation treatment positively influences moisture-retention capacity. From a molecular perspective, the hydroxyl groups within the cellulose framework serve as the primary sites for hydrogen-bonding with water molecules. The irradiation treatment may induce the partial degradation of cellulose chains, alterations in crystallinity, and increased exposure of hydroxyl groups, consequently enhancing the material’s affinity for hydrogen-bonding with water molecules. The augmentation in hydroxyl functionality and their enhanced accessibility facilitates the formation of a stable hydrogen-bonded network upon interaction with water molecules, consequently diminishing the rate of water loss under external mechanical stress. Consequently, the exceptional water-retention capacity of IRSC–HWAG can be predominantly ascribed to the abundance of hydroxyl groups within the cellulose matrix and their robust hydrogen-bonding interactions with water molecules [49]. The expanded network architecture exerts a decisive influence on the material’s water absorption properties. Structural modifications induced by irradiation may engender a more open pore distribution and an augmented water-capture capability within the cellulose network, thereby achieving superior water-retention performance under equivalent external pressure conditions. Furthermore, water diffusion dynamics within the network are modulated by several factors, including pore size distribution, cross-link density, and the thickness of water layers [50].

## 3. Conclusions

This process obviates the necessity of chemical reagents while fundamentally altering the recalcitrant native architecture of cellulose, directly reducing its degree of polymerization and crystallinity. The procedure exhibits remarkable operational simplicity and controllability, with the extent of degradation being precisely tunable through adjustment of the irradiation dose, a critical parameter typically ranging from 48 to 144 kGy. Consequently, this methodology yielded cellulose derivatives exhibiting enhanced solubility and heightened reactivity.

Through systematic single-factor and orthogonal experimental analyses, the following optimal conditions were established: the monomer ratio of acrylic acid (AA) to acrylamide (Am) was 1.5:1, the material ratio of (AA + Am) to irradiated rice straw (IRS) was 9:1, and the neutralization degree of acrylic acid reached approximately 90%. The irradiation parameters were set at a dose of 5 kGy with a dose rate of 2.0 kGy/h, while the crosslinking agent concentration was maintained at 1.2%. Under these optimized conditions, the resultant grafted copolymer exhibited a water absorption capacity of approximately 1160 g/g and a saline water absorption rate of approximately 99 g/g.

## 4. Materials and Methods

### 4.1. Main Raw Materials, Reagents, and Equipment

Reed Straw (RS); acrylamide (analytical pure, Am), acrylic acid (analytical pure, AA); sodium hydroxide (analytical pure); urea (analytical pure); N, N′-methylene bisacrylamide (analytical pure, MBA); anhydrous ethanol (analytical pure); N, N–dimethylacetamide (analytical pure, DM), lithium chloride (analytical pure, LiCl). All agents were sourced from Shanghai Chemical Reagent Corporation (Shanghai, China); Reed samples were collected along the bank of the Tuo River in Suzhou City, Anhui Province.

Fourier transform infrared spectrometer (S-200, Nicolet Company, Madison, WI, USA); electric heating constant-temperature blast-drying box (DHG-9070: Shanghai Jinghong Experimental Equipment Co., Ltd., Shanghai, China); thermostatic water bath (R2017: Shanghai Guangying Instrument Co., Ltd., Shanghai, China); electronic analysis balance (FA2104N: Shanghai Qinghai Instrument Co., Ltd., Shanghai, China); thermogravimetric analyzer (HTG-1, Beijing Hengjiu Scientific Instrument Company, Beijing, China); ^60^Co γ-radiation source, (National) Forestry Irradiation Center (Hefei, China), with an activity of 200,000 curies; a single-grid plate was used; absorptiometry was measured using dichromate dosimeter calibration.

### 4.2. Preparation of the Dissolution Solution

(A) Preparation and Dissolution of LiCl/DMAC Solution

Introduce rigorously dried lithium chloride (LiCl) into anhydrous N, N-dimethylacetamide (DMAC), ensuring the former has been subjected to thorough drying—typically via prolonged oven desiccation followed by equilibration in a desiccator—while the latter must be meticulously dehydrated, for instance, through treatment with molecular sieves. Subsequently, heat the mixture to 90 °C with continuous agitation until a completely transparent solution is achieved. It is imperative that the resulting LiCl/DMAC solvent system itself is prepared in advance and maintains anhydrous conditions throughout.

For the dissolution procedure, incorporate the DMAC-swollen cellulose, obtained from the preceding preparative step, into the prepared 8% (*w*/*v*) LiCl/DMAC solution. Subject this mixture to thermal treatment at 90 °C under vigorous mechanical stirring for a defined duration, which may range from several minutes to multiple hours, contingent upon the origin of the cellulose substrate and its degree of polymerization. Subsequently, reduce the system’s temperature to ambient conditions or employ an ice-water bath, maintaining agitation for an extended period (typically 20 h). This cooling phase is of critical importance, as the formation and subsequent dissolution of the RS–LiCl complex proceed with greater thermodynamic stability and completeness at reduced temperatures. The ultimate product is a limpid, highly viscous, and gel-free cellulose solution.

(B) Preparation of Artificial Urine

The formula for each liter of artificial urine is as follows (can be scaled up or down as needed): urea 2.0 g; sodium chloride (NaCl) 9.0 g; potassium chloride (KCl) 0.5 g; ammonium bicarbonate (NH_4_Cl) 0.5 g.

(C) Preparation of Normal Saline

A total of 0.9% NaCl (g/g) is used; weigh 9 g of salt and add it to 993 of deionized water to prepare a 0.9% salt solution.

### 4.3. Pre-Treatment of RS for De-Crystallization

(A) Preparation of Reed Straw Substrate

Reed stalks were meticulously segmented into 3 cm segments and subsequently subjected to mechanical pulverization using a grinding apparatus (TRS-800g-N01, TERUISI, Foshan, Guangdong, China). The resulting particulate material was then passed through a 60-mesh sieve to obtain uniform reed straw (RS) particles.

- Alkaline/Urea Dissolution Process

For the dissolution procedure, 20 g of RS powder was combined with 24 g of sodium hydroxide (NaOH) and 16 g of urea in 360 mL of deionized water, yielding a 6% (*w*/*v*) NaOH/4% (*w*/*v*) urea aqueous solution. The mixture was thoroughly homogenized and subjected to depolymerization at a controlled temperature of 25 °C for a duration of 3 h with continuous agitation. Subsequently, the product was separated via centrifugation and subjected to repeated washing with distilled water until the complete absence of chloride ions (Cl^−^) was confirmed through silver nitrate (AgNO_3_) precipitation testing. The resulting purified reed straw cellulose (RSC) was designated as sample C1.

b. LiCl/DMAC dissolution process

Five percent (*w*/*w*) of reed straw (RS) powder was introduced into an 8% (*w*/*w*) lithium chloride/N, N-dimethylacetamide (LiCl/DMAC) solvent system within three separate test tubes. The mixture was subjected to vigorous agitation at ambient temperature for a 24 h period using a magnetic stirrer, resulting in the formation of a dark, clear, and transparent RS/LiCl/DMAC solution. Subsequently, this solution was gradually introduced dropwise into a fivefold volume of distilled water with continuous stirring. Following this, the mixture was permitted to undergo a period of sedimentation. The resultant product was isolated via centrifugation and subsequently washed with distilled water until the complete absence of chloride ions (Cl^−^) was confirmed through silver nitrate (AgNO_3_) testing. The prepared cellulose derivative was designated as C2.

(B) Preparation of Irradiated Reed Straw Cellulose (IRSC)

Reed stalks were precisely sectioned into 3 cm segments and positioned within an irradiation facility. The specimens were subjected to irradiation at a dose rate of 2.0 kGy/hour for successive durations of 24, 48, and 72 h, corresponding to total radiation exposures of 48, 96, and 144 kGy, respectively. Following irradiation, the material was pulverized using a mechanical grinder and subsequently passed through a 60-mesh sieve to obtain uniformly sized particles. The final product was irradiated reed straw cellulose (IRSC).

- NaOH/Urea Dissolution Process

The dissolution of IRS utilizing a NaOH/urea aqueous system was executed through the following standardized protocol. Initially, 20 g of IRS powder was introduced into a solution comprising 360 mL of deionized water, 24 g of NaOH, and 16 g of urea, thereby preparing a mixture with concentrations of 6 wt% NaOH and 4 wt% urea. The resultant suspension was subjected to continuous agitation to ensure the formation of a homogeneous solution, subsequently maintained at 25 °C to facilitate the depolymerization of IRS. Following a 3 h period of stirring, the product was isolated via centrifugation. The supernatant was then subjected to exhaustive washing with distilled water until the complete absence of chloride ions (Cl^−^) was confirmed through AgNO_3_ testing. The resulting IRSC products were systematically designated as C3 (48 kGy), C5 (96 kGy), and C7 (144 kGy) for subsequent analysis.

b. LiCl/DMAC Dissolution Procedure

The IRS powder was meticulously dispersed within an 8% (*w*/*w*) lithium chloride in dimethyl sulfoxide (DMSO) solvent system to achieve a final IRS concentration of 5% (*w*/*v*). The resulting suspension underwent continuous mechanical agitation at ambient temperature for a 24 h period utilizing a magnetic stirrer, ultimately yielding a dark, limpid, and transparent IRS/LiCl/DMSO homogeneous solution. Subsequently, this solution was introduced dropwise into distilled water in five aliquots of equal volume, accompanied by vigorous agitation to ensure thorough homogenization, and permitted to stand for a designated period. Following centrifugation to recover the precipitated product, the solid was subjected to repeated washing with distilled water until the complete absence of chloride ions was confirmed by silver nitrate (AgNO_3_) testing. The resultant IRS products were systematically designated as C4 (48 kGy), C6 (96 kGy), and C8 (144 kGy).

### 4.4. HWAG Preparation of Different Treated Lignocellulose Graft Copolymer Gels

Weigh a predetermined quantity of prepared IRSC and reed straw cellulose (RSC) powders into a dry, immaculate glass vessel. Gradually introduce the corresponding volume of deionized water while agitating the mixture until a limpid solution is achieved. Simultaneously, subject a measured portion of acrylic acid (AA) to refrigeration in a water bath, followed by its incremental addition to a sodium hydroxide solution of specified molar concentration to yield the AA-containing solution. In accordance with the experimental protocol, prepare the IRSC and RSC powders along with a polymerization inhibitor (FeSO_4_·7H_2_O). Combine all aforementioned constituents to form a homogeneous system, employing mechanical stirring and ultrasonic dispersion as necessary, subsequently subjecting the mixture to irradiation to synthesize the rubber gel resin. The resulting resin is desiccated at 60 °C, subsequently pulverized, and subjected to exhaustive washing with anhydrous ethanol, allowing the solvent to volatilize completely, followed by final drying. The crude product is then subjected to thermal treatment at 70 °C, ground to a fine powder, and extracted with acetone in a Soxhlet apparatus for 24 h to eliminate any residual homopolymers.

Following complete acetone evaporation, the specimens are desiccated at 50 °C until constant mass is achieved, thereby yielding the purified graft copolymer. Subsequent to the crystallization-removal pretreatment, the synthesized IRSC and RSC powders are designated as C1 through C8, corresponding respectively to the highly hygroscopic gels HWAG1 through HWAG8. All experimental procedures were conducted in triplicate to ensure statistical reliability.

In an alternative synthetic pathway, a predetermined quantity of acrylic acid is subjected to cooling in a water bath prior to gradual introduction into a sodium hydroxide solution of specified mass fraction. Subsequently, acrylic acid, acrylamide (Am), the crosslinking agent N, N′-methylene bisacrylamide (MBA), and the polymerization inhibitor ferrous sulfate heptahydrate (FeSO_4_·7H_2_O) are incorporated. The resultant mixture is thoroughly agitated to ensure homogeneity, followed by irradiation within the specialized irradiation facility. The crude product is initially desiccated at 70 °C, and then subsequently pulverized and subjected to Soxhlet extraction with acetone for a 24 h period to eliminate any homopolymer constituents. Following complete acetone evaporation, the material is dried at 50 °C until constant mass is attained, yielding the purified polymer corresponding to HWAG. All experiments are repeated three times.

### 4.5. Determination of the Sample

(1) Water Absorption Measurements of HWAG

A specified amount of AA is cooled in a water bath and slowly added to a sodium hydroxide solution of a given mass fraction. The water absorption capacity is evaluated at room temperature. A 0.5 g sample is separately submerged in distilled water and saline solution to induce swelling. Excess moisture is removed with a 120-mesh sieve. The swollen mass is recorded until no further liquid drips from the sample. Each sample is tested three times, and standard errors for Q are calculated from covariance (ANCOVA) using SPSS v. 19.0 (IBM Corp., Armonk, NY, USA).

The water absorption rate is determined using Equation (1):

(1) $$Q=\frac{m_{2}-m_{1}}{m_{1}}\times 100\%$$

The water absorption rate (Q, g/g) for the HWAG is computed using the specified formula [3]. *m*_1_ represents the dry sample’s quality and *m*_2_ represents the quality of the swollen sample (g). Q is expressed as grams of water per gram of sample (g/g).

In our study, several parameters are used to measure water absorption capacity: the quality of distilled water (Q_d_, g/g), the quality of tap water (Q_t_, g/g), the sodium chloride solution’s quality (Q_s_, g/g), and the quality of the artificial urine (Q_a_, g/g).

(2) Determination of Water-Retention Properties of Gels

Prepare a gelatinous matrix comprising uniformly sized particles within the 100–200 mesh range. Precisely weigh 0.2 g aliquots of the gel into multiple 1500 mL beakers. Subsequently, introduce 1000 mL of deionized water to each beaker and maintain agitation to ensure homogeneity. Following this, remove the aqueous medium through filtration employing a nylon mesh with a pore diameter of 0.154 mm. Thereafter, determine the mass of the hydrated specimens after exposure to thermal treatment at 60 °C for varying durations (0, 20, 40, 60, 80, and 100 h). This experimental protocol facilitates the establishment of a quantitative relationship between water absorption capacity and elapsed time, as delineated in Equation (2) for the gel water-retention test.

(2) $$R=\frac{m_{h}}{m}\%$$

with *m_h_* representing the heated sample’s quality at different times, and *m* representing the quality of the control swollen sample (g). *R* represents the percentage of water retention.

### 4.6. Product Characterization

- FTIR spectroscopy was performed on a Thermo Nicolet NEXUS instrument (Thermo Fisher Scientific Inc, Madison, WI, USA), with sample pressing using potassium bromide.
- A morphological analysis was conducted with a JEOL JSM-5600LV scanning electron microscope (JEOL Ltd., Tokyo, Japan). HWAG powder passed through a 200-mesh sieve was examined at 500× magnification in a dry state, and samples were coated with a thin gold film at an accelerating voltage of 15 kV.
- Thermal stability was evaluated by thermogravimetric analysis (TGA) using a Netzsch STA 449C instrument (NETZSCH, Selb, Bavaria, Germany). Measurements were carried out from 25 to 550 °C at a heating rate of 10 °C/min under a dry nitrogen flow of 40 mL/min.

### 4.7. Image Generation and Data Processing

During the preparation of this manuscript, the authors used ChatGPT 4.0 for the purposes of Figure 1. This picture is a schematic diagram illustrating the principle of strong irradiation-induced degradation of cellulose, and it is not experimental data or analysis. The authors have reviewed and edited the output and take full responsibility for the content of this publication. Figure 2, Figure 3 and Figure 7, were processed using origin2024; Figure 5 and Figure 6, Table 1, Table 2 and Table 3 were processed using SPSS v. 19.0 (IBM Inc., Armonk, NY, USA).

## Figures and Tables

**Table 1:** Different pre-treatment yields of reed straw cellulose.

Sample	Solvent System	Dose (kGy)	Yield (%)
C1	NaOH/urea	0	14 ± 1 ^d^
C2	LiCl/DMSO	0	18 ± 2 ^c^
C3	NaOH/urea	48	22 ± 3 ^b^
C4	LiCl/DMSO	48	25 ± 2 ^a^
C5	NaOH/urea	96	27 ± 2 ^a^
C6	LiCl/DMSO	96	30 ± 3 ^a^
C7	NaOH/urea	144	27 ± 2 ^a^
C8	LiCl/DMSO	144	27 ± 3 ^b^

Note: Different letters within a line represent significant differences (*p* < 0.05).

**Table 2:** The effect of different RSC preparation methods on the HWAG’s absorbency.

Sample	Absorbency			
	Quality of Distilled Water (Q_d_, g/g)	Quality of Tap Water (Q_t_, g/g)	Sodium Chloride Solution (Q_s_, g/g)	Artificial Urine (Q_a_, g/g)
HWAG 1	655 ± 20 ^f^	226 ± 12 ^d^	56 ± 2 ^f^	56 ± 2 ^e^
HWAG 2	701 ± 31 ^e^	224 ± 13 ^d^	64 ± 3 ^e^	55 ± 3 ^e^
HWAG 3	761 ± 21 ^d^	356 ± 12 ^c^	71 ± 3 ^d^	63 ± 2 ^d^
HWAG 4	852 ± 31 ^c^	424 ± 13 ^b^	79 ± 2 ^c^	64 ± 3 ^cd^
HWAG 5	1085 ± 24 ^b^	571 ± 13 ^a^	82 ± 5 ^b^	69 ± 3 ^b^
HWAG 6	1280 ± 27 ^a^	575 ± 15 ^a^	92 ± 5 ^a^	79 ± 3 ^a^
HWAG 7	855 ± 24 ^c^	452 ± 12 ^c^	81 ± 2 ^b^	66 ± 2 ^c^
HWAG 8	841 ± 32 ^c^	424 ± 13 ^b^	81 ± 2 ^b^	65 ± 3 ^c^

Note: Different letters within a line represent significant differences (*p* < 0.05). Quality of water: the amount of water absorbed by each gram of the colloid.

**Table 3:** Orthogonal experiment results.

	A m_m_:m_IRSC_ (g/g)	B Neutralization of AA (%)	C Dose (kGy)	D m_AA_:m_Am_ (g/g)	Q_d_ in the Distilled Water (g/g)	Q_s_ in the Saline Solution (g/g)
1	1 (7:1)	1 (70)	1 (4)	1 (1.2)	586 ± 28	58 ± 4
2	1	2 (80)	3 (5)	2 (1.5)	885 ± 32	68 ± 3
3	1	3 (90)	2 (6)	3 (1.8)	789 ± 35	75 ± 2
4	2 (8:1)	1	3	3	892 ± 39	79 ± 3
5	2	2	2	1	1124 ± 34	85 ± 2
6	2	3	1	2	1254 ± 29	89 ± 2
7	3 (9:1)	1	2	2	1245 ± 21	95 ± 3
8	3	2	1	3	957 ± 28	94 ± 2
9	3	3	3	1	854 ± 31	85 ± 3
${k_{1}}^{a}$	753 ± 31	907 ± 29	932 ± 28	855 ± 31		
$k_{2}$	1090 ± 34	989 ± 31	1053 ± 30	1128 ± 27		
$k_{3}$	1018 ± 27	966 ± 32	877 ± 34	879 ± 34		
$R^{b}$	337	82	176	273		
${k_{1}^{\prime }}^{c}$	67 ± 4	77 ± 3	80 ± 4	76 ± 3		
$k_{2}^{\prime }$	84 ± 4	82 ± 5	85 ± 3	84 ± 3		
$k_{3}^{\prime }$	91 ± 3	83 ± 4	77 ± 4	83 ± 3		
${R^{\prime }}^{d}$	24	6	8	8		
Q	A_3_	B_3_	C_2_	D_2_		
k1a = (Σ the water absorbency in the distilled water of single-factor)/3						
R1b=maxk1−mink1						
k1′a = (Σ the water absorbency in saline solution of single-factor)/3						
R1d=maxk′1−mink′1						
Q = the optimum conditions						

## Data Availability

The data generated during the present study are available from the corresponding authors upon reasonable request.
